# OP22 Artificial-Intelligence-Powered Prompts For Simplified Cost-Effectiveness And Net-Benefit Analysis In Health Technology Assessment

**DOI:** 10.1017/S0266462325100809

**Published:** 2025-12-29

**Authors:** Bruno Barros, Carlos Alberto Magliano, Quenia Cristina Dias Morais, Marcelo Correia, Bernardo Tura

## Abstract

**Introduction:**

Health technology assessment (HTA) often involves complex cost-effectiveness analyses (CEA) that can be challenging for non-experts. This study demonstrated the use of artificial-intelligence (AI)-powered prompts to simplify CEA processes. By integrating tools for generating efficiency frontiers and net-benefit analyses, this approach enables stakeholders, such as non-modeling HTA specialists or decision-makers, to understand therapeutic scenarios and make informed adjustments to analyses.

**Methods:**

The prompts were created using ChatGPT-4o and tested for usability and reproducibility in ChatGPT-4o and the free version 3.5. The prompts were designed to automate key steps in economic analysis, including calculating net monetary and health benefits, performing cost-effectiveness analyses applying dominance and extended dominance concepts, and generating the efficient frontier plot. Ten HTA experts with no modeling experience evaluated the prompts using predefined scenarios with hypothetical datasets. The results of both versions of ChatGPT were compared to the expected results. The usability and accuracy of the prompts were assessed during the evaluation.

**Results:**

ChatGPT-4o achieved 100 percent accuracy in calculating net health and monetary benefits (NHB and NMB) and correctly applied the concepts of dominance and extended dominance in 83 percent of cases. It calculated the incremental cost-effectiveness ratio (ICER) for non-dominated therapies in 50 percent of situations and successfully generated the efficiency frontier graphic in half of cases. In contrast, ChatGPT 3.5 achieved 50 percent accuracy for NHB and NMB calculations, only 17 percent for applying dominance and extended dominance concepts, and failed to calculate ICERs or generate the efficiency frontier plot as expected.

**Conclusions:**

AI-powered prompts simplified cost-effectiveness analyses by enabling non-technical stakeholders to create and adjust efficiency frontiers and benefit analyses. ChatGPT-4o demonstrated improved reliability. Limitations in ChatGPT 3.5, particularly with ICER calculation and graphical outputs, indicated the need to adapt prompts to specific AI tools. Future developments may increase the robustness and usability of these types of prompts across platforms.

